# Influence of Base Plate Preheating on Laser Powder Bed Fusion–Processed EN AW-7075 Aluminium Alloy

**DOI:** 10.3390/ma19050970

**Published:** 2026-03-03

**Authors:** Nejc Velikajne, Jožef Medved, Črtomir Donik, Irena Paulin

**Affiliations:** 1 Institute of Metals and Technology, Lepi pot 11, SI-1000 Ljubljana, Slovenia; nejc.velikajne@imt.si (N.V.); crtomir.donik@imt.si (Č.D.); 2 Faculty of Natural Sciences and Engineering, University of Ljubljana, Aškerčeva cesta 12, SI-1000 Ljubljana, Slovenia; jozef.medved@ntf.uni-lj.si

**Keywords:** laser powder bed fusion (LPBF), EN AW 7075, cracks, solidification rate, base plate preheat

## Abstract

The influence of base plate temperature (25, 100, 200, and 400 °C) on the laser powder bed fusion processing of EN AW 7075 was systematically investigated using microstructural characterisation (LM, SEM, EBSD, GROD), chemical analysis, hardness testing, and thermal simulations across a broad range of process parameters. Moderate preheating at 100 °C and 200 °C showed no significant reduction in crack density or changes in grain morphology compared to processing without preheating. At the highest studied temperature—400 °C—a transition to columnar crack networks was observed, accompanied by modified grain orientation, pronounced stress relaxation, and reduced hardness. Independent of preheating temperature, consistent evaporation of Zn (~1 wt.%) and Mg (~0.3 wt.%) occurred during processing. Thermal simulations qualitatively supported the experimental observations, indicating increased thermal retention and displacement with increasing preheating temperature. The results demonstrate that base plate preheating alone is insufficient to suppress hot cracking in EN AW 7075 and may promote alternative crack-growth mechanisms at elevated temperatures, highlighting the need for alternative alloy or process design strategies.

## 1. Introduction

Laser powder bed fusion (LPBF) has matured into a leading metal additive manufacturing (AM) technology for producing complex near-net-shape parts with high geometric resolution and the potential for microstructure tailoring. Aluminium alloys are particularly attractive for LPBF because of their low density and high specific strength; however, industrial use remains dominated by castable Al–Si alloys (e.g., AlSi10Mg) that exhibit comparatively good weldability and reduced cracking tendency under rapid solidification conditions [1,2,3]. In contrast, high-strength heat-treatable aluminium alloys of the 7xxx series—especially EN AW 7075 (Al–Zn–Mg–Cu)—remain among the most difficult Al alloys to process by LPBF due to severe solidification (hot) cracking and the volatility of key alloying elements [4,5,6,7,8,9,10].

The susceptibility of 7xxx alloys to hot cracking is rooted in fundamental solidification metallurgy: a relatively wide solidification temperature range, strong microsegregation, and limited interdendritic liquid feeding under tensile strain in the semi-solid temperature interval [11,12]. In LPBF, steep thermal gradients and high cooling rates promote epitaxial columnar grain growth and non-equilibrium solidification, creating long intergranular liquid films and crack paths along grain boundaries and melt pool boundaries [4,5,13]. For EN AW 7075, multiple studies [9,10] have reported persistent crack networks even when porosity is reduced through parameter optimisation, indicating that cracking is controlled primarily by intrinsic alloy-solidification behaviour rather than by energy density alone.

Beyond cracking, LPBF processing of EN AW 7075 is complicated by preferential evaporation of Zn and Mg from the melt pool [9,13,14,15]. These elements have comparatively high vapour pressures at laser-induced temperatures, and their loss can alter phase equilibria, precipitation kinetics, and strengthening response, while also influencing solidification behaviour and defect formation [10,16,17,18,19]. Recent laser-processing work continues to emphasise that selective Zn/Mg evaporation can measurably change local chemistry and mechanical response in AA7075, underlining the importance of quantifying composition shifts in LPBF-related thermal cycles [7,9,10].

Accordingly, a considerable amount of recent research has focused on strategies to improve the printability of Al7075/7xxx alloys [15,20,21]. Approaches based on alloy and feedstock modification—including inoculation and grain-refining additions—have shown that transforming coarse columnar grains into fine equiaxed grains can markedly reduce or eliminate hot cracks. For example, crack suppression has been demonstrated through engineered heterogeneous nucleation routes and in situ formation of nucleating phases (e.g., TiB_2_/Al_3_Ti-type mechanisms) [22,23], through powder-blending approaches that refine grains via intermetallic-assisted nucleation [24], and through mechanical-alloying-assisted inoculation routes enabling crack-free LPBF 7075 [25]. Complementary “materials-by-design” work published in 2023 proposed integrated computational–experimental composition modifications (grain refiners and eutectic solidification enhancement) to improve LPBF processability of 7xxx alloys while limiting defect formation [26]. In parallel, broader critical reviews published in 2025 consolidate rapid growth in AM research for Al7xxx [9] systems and highlight that cracking and element evaporation remain central barriers to scalable, reliable manufacturing.

In addition to chemistry-based strategies, thermal management—particularly base plate preheating—remains an industrially attractive option because it can reduce thermal gradients, slow cooling rates, and potentially reduce residual stress accumulation without altering feedstock chemistry [27,28]. For Al7075, experimental work [29] has shown that elevated base plate temperatures can reduce the extent of porosity [4] and cracking [24], but it typically does not guarantee crack-free fabrication across practical processing windows and may interact with microsegregation and crack propagation mechanisms [30]. Thermo-mechanical modelling [31,32] frameworks have also demonstrated that preheating strongly affects thermal history, distortion, and stress evolution in LPBF builds, motivating combined experimental–simulation studies for process optimisation and defect control [3,28,33,34]. In the present work, thermo-mechanical LPBF simulations were performed using Autodesk Netfabb to evaluate the influence of base plate preheating on temperature distribution and displacement in EN AW 7075 builds. Simulations were conducted for preheating temperatures of 25, 100, 200, and 400 °C using the same cube geometry and processing sequence as in the experiments. The modelling approach provides comparative trends in thermal retention and distortion with increasing preheating temperature, enabling direct correlation with experimentally observed crack morphology and hardness evolution.

Despite these advances, there remains a need for systematic experimental evidence linking (i) preheating temperature, (ii) chemical stability of Zn/Mg during LPBF, (iii) crack morphology and grain structure evolution, and (iv) local lattice misorientation/internal strain metrics (e.g., EBSD-based GROD) and hardness response in EN AW 7075 processed under controlled LPBF conditions. Such integrated datasets are essential for clarifying whether preheating primarily mitigates cracking by reducing thermal stresses, or whether it may promote alternative crack-growth pathways through changes in melt pool lifetime, segregation behaviour, and stress relaxation.

Therefore, the present study, with the central hypothesis that increasing the base plate temperature reduces thermal gradients and solidification stresses sufficiently to mitigate hot cracking in EN AW 7075, is conducted. By systematically varying the preheating temperature from 25 to 400 °C, this work aims to identify its influence on preheating when it becomes ineffective or even detrimental. By investigating four discrete base plate preheating temperatures (25, 100, 200, and 400 °C), this work aims to identify the thermal threshold beyond which preheating becomes ineffective or detrimental. The chosen temperatures were selected to represent typical LPBF conditions (25 °C), moderate preheating commonly applied in practice (100–200 °C), and elevated preheating near the upper range reported in the literature for crack mitigation studies (400 °C). Hardness measurements were correlated with local strain characteristics, and thermo-mechanical simulations based on inherent strain were performed to evaluate thermal distributions and displacements as a function of preheating temperature.

## 2. Materials and Methods

The EN AW 7075 aluminium powder was obtained from Shanghai Truer Technology Co. (Shanghai, China). Table 1 shows the chemical composition of the EN AW 7075 powder. The chemical composition of the powder alloy was determined using inductively coupled plasma (ICP) analysis.

Powder morphology and particle size distribution were evaluated because particle shape and size characteristics directly influence powder bed packing density, flowability, and laser–material interaction, which in turn affect melt pool stability and defect formation during LPBF. Morphology was analysed with a ZEISS Crossbeam 550 (Carl Zeiss GmBH, Oberkochen, Germany) Scanning Electron Microscope (SEM), which is equipped with Energy-Dispersive X-ray Spectroscopy (EDS). SEM analysis provided high-resolution images of the aluminium powder particles, revealing the morphology and particle size.

Particle size distribution was measured using a Horiba LA-920 (Horiba Ltd., Kyoto, Japan) laser scattering analyser. Samples were manufactured using an AconityMINI (Aconity3D GmbH, Herzogenrath, Germany) LPBF system. Laser power and scanning speed were systematically varied to evaluate their effects on the fabricated samples. Samples had a cube geometry (10 × 10 × 10 mm^3^).

All selected parameters were derived from a preliminary process map, machine manufacturer, and found in the literature [35,36]. Table 2 presents the parameters used for manufacturing. Samples are arranged by energy density from lowest to highest The laser power (P) values varied between 150 W and 400 W, the scanning speed (v) values varied from 600 mm/s to 1400 mm/s, laser beam diameter (d) values were set to 60 μm, hatch distance (h) was obtained at 60 μm, and the layer thickness (l = 30 µm) was kept constant. The energy density (E) was calculated using the equation:

(1) $$E=\frac{P}{v*d*l*h}$$

A ZEISS Axio Imager.Z2m (Carl Zeiss GmBH, Oberkochen, Germany) was used for light microscopy (LM). SEM EBSD analyses were performed on a ZEISS FIB-SEM CrossBeam 550 with an EDAX Hikari super EBSD Camera (EDAX, Inc., Mahwah, NJ, USA). Electron Backscatter Diffraction-based Grain Reference Orientation Deviation (EBSD-GROD) quantifies the local lattice misorientation of each point relative to a reference orientation within the same grain. It is commonly used to visualise and assess intragranular plastic deformation and strain heterogeneity at the microscale. All microscopy results are presented in a coordinate system in which the vertical axis corresponds to the build direction.

**Figure 1:**
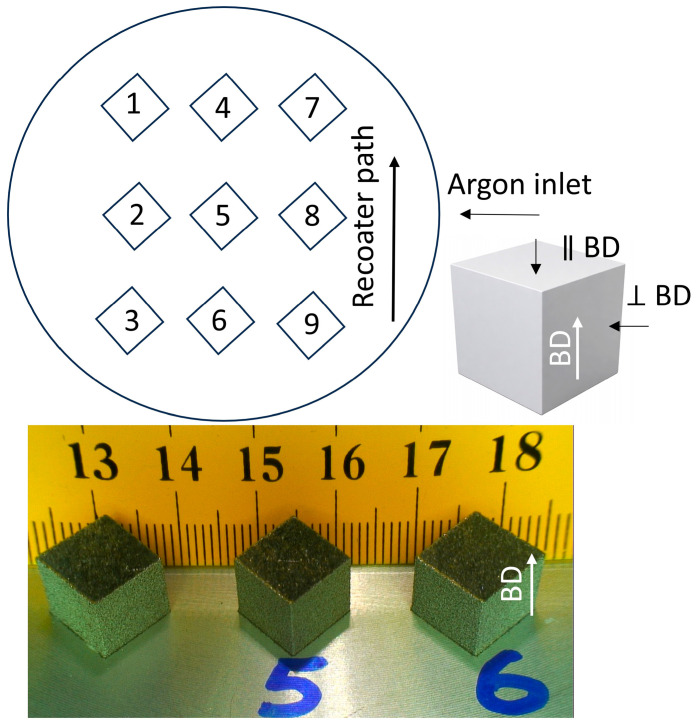
Experimental setup with samples placed on the build plate and fabricated samples.

Figure 1 shows the LPBF experimental setup with sample placement on the build plate and recoater direction (top), together with representative fabricated cube specimens (bottom).

All samples were analysed by microscopy along sections parallel to the build direction (bottom–top orientation).

Vickers hardness measurements (HV0.5) were conducted on LPBF-fabricated EN AW 7075 samples produced with powder bed preheating temperatures of 25, 100, 200, and 400 °C. For each condition, three indentations were performed on each sample. Hardness measurements were carried out on polished cross-sections, both perpendicular and parallel to the build direction, as shown in the scheme in Figure 1, in order to account for possible microstructural anisotropy induced by the LPBF process. Indentations were placed in the central region of the specimens, away from edges and visible cracks, to ensure a representative bulk material response. The average hardness values are summarised in Table 4.

Thermal simulations of the LPBF process were performed using Autodesk Netfabb Premium (Autodesk Inc., San Francisco, CA, USA), employing the inherent strain-based thermo-mechanical simulation module for additive manufacturing. The simulated geometry corresponded to the cubic specimens (10 × 10 × 10 mm^3^) used in the experiments. Base plate preheating temperatures of 25, 100, 200, and 400 °C were applied as initial thermal boundary conditions. The base plate was modelled as rigidly fixed, while heat transfer to the surrounding environment was represented by a convective boundary condition at the free surfaces. Temperature-dependent thermophysical properties of EN AW 7075 were taken from the built-in Netfabb material database for aluminium alloys. The powder-to-solid transition was treated using an effective material approach typical for inherent-strain LPBF simulations. The computational mesh consisted of voxel-type elements with uniform resolution across the part and a refined representation near the base plate interface. Layer-wise activation was applied to represent the additive build sequence. A mesh sensitivity analysis was performed to ensure that the predicted temperature and displacement fields were independent of element size. Simulations were conducted using three voxel mesh resolutions (coarse: 0.4 mm, medium: 0.25 mm, fine: 0.15 mm). The resulting maximum temperature and displacement values differed by less than 3% between the medium and fine meshes, indicating numerical convergence. Based on this analysis, the medium mesh resolution (0.25 mm voxel size) was adopted for all simulations to balance accuracy and computational efficiency. Similar mesh convergence approaches have been reported in recent thermo-mechanical modelling studies of additive manufacturing processes [37].

## 3. Results

**Figure 2:**
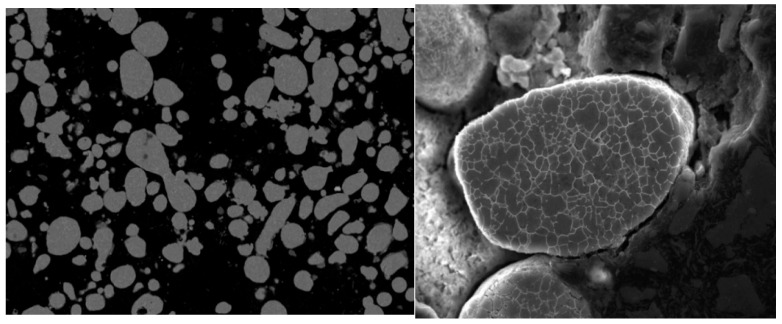
SEM images of EN AW 7075 powder morphology showing predominantly spherical particles.

**Figure 3:**
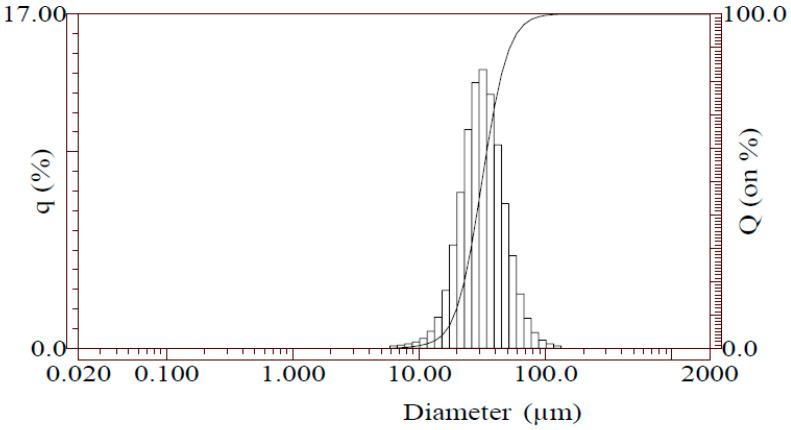
Particle size distribution obtained by laser diffraction, presented as cumulative distribution (Q) and distribution density (q).

The powder particles were semi-spherical and ranged in size from 10 µm to 100 µm. The powder morphology is shown in Figure 2. The particles’ surfaces appeared relatively smooth, indicative of a well-prepared, atomised powder. Particle size percentiles D10 and D50 were evaluated, representing the particle diameters below which 10% and 50% of the cumulative particle volume are contained, respectively. The D10 value was estimated to be approximately 8 µm. The cumulative distribution curve suggests a D50 of approximately 50–60 µm, as indicated by the midpoint of the percentage scale on the graph (Figure 3). Based on the cumulative distribution curve, the D50 value was visually estimated to be approximately 50–60 µm.

Table 3 presents the chemical analysis results for four LPBF-fabricated samples. Each sample underwent ICP analysis to assess its elemental composition. The findings reveal the influence of the LPBF process on the material’s chemistry. Notably, the analysis highlights variations in Zn and Mg composition across the samples. The results indicate the evaporation of Zn and Mg during LPBF, leading to chemical compositions that differ from those of the initial powder. This phenomenon underscores the dynamic nature of the LPBF technique, whereby high-energy laser interactions can volatilize certain elements. Consequently, the printed samples exhibit altered chemical compositions, particularly in terms of Zn and Mg content. The mass fraction of Zn in all samples was reduced by approximately 1 wt.% and the mass fraction of Mg by 0.3 wt.%. These findings extend the understanding of the mechanisms that drive material alterations during additive manufacturing, with implications for material tailoring and process consistency.

### 3.1. Microscopic Observations

**Figure 4:**
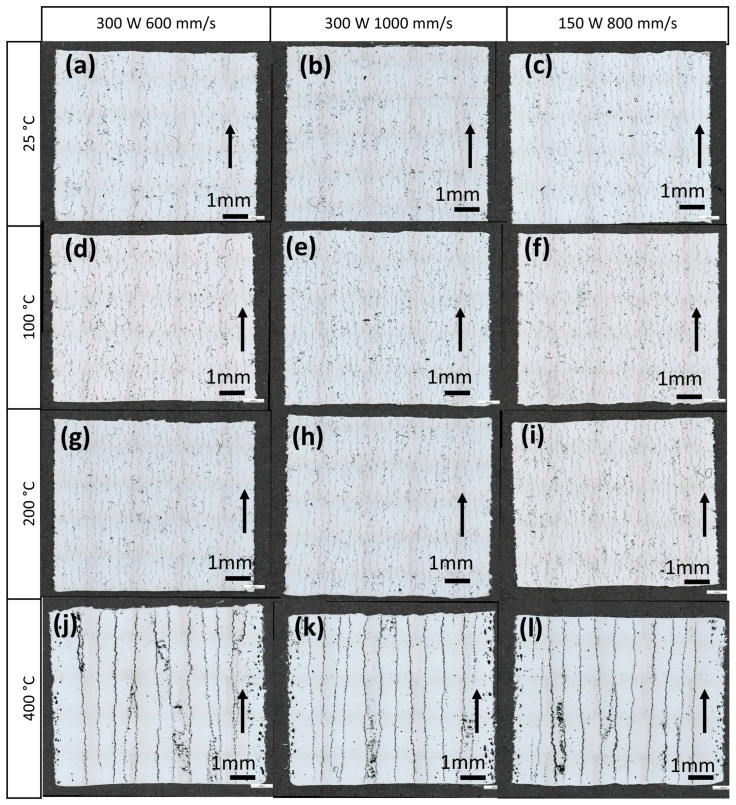
LM images of LPBF-produced samples with varied process parameters and preheating temperatures ranging from 25 to 400 °C.

Figure 4 illustrates a comprehensive examination of 3D-printed samples. LM was used to capture microstructures at the macroscopic level. The printed samples were processed with laser power ranging from 150 W to 400 W and scanning speeds from 600 to 1400 mm/s, with variations in preheating temperatures (no preheating, 100, 200, and 400 °C). The directional build process is indicated by black arrows. A prominent observation from the figure is the presence of conspicuous hot cracks within the microstructures’ cross-sections. These cracks have developed during the printing process, underscoring the significance of defect formation. To mitigate these defects, a strategy of reducing cooling speed was proposed by elevating the base plate temperature. Samples printed at 100 °C and 200 °C exhibit microstructural characteristics comparable to those printed under room temperature conditions. A distinct microstructural change was observed in samples printed at 400 °C. Here, an interesting phenomenon of columnar cracks becomes evident in LM images. This unique effect indicates a complex interaction between temperature gradients, material solidification, and structural integrity during additive manufacturing. Figure 3 provides a compelling visual representation of how preheating temperatures influence microstructural features, especially concerning crack development.

**Figure 5:**
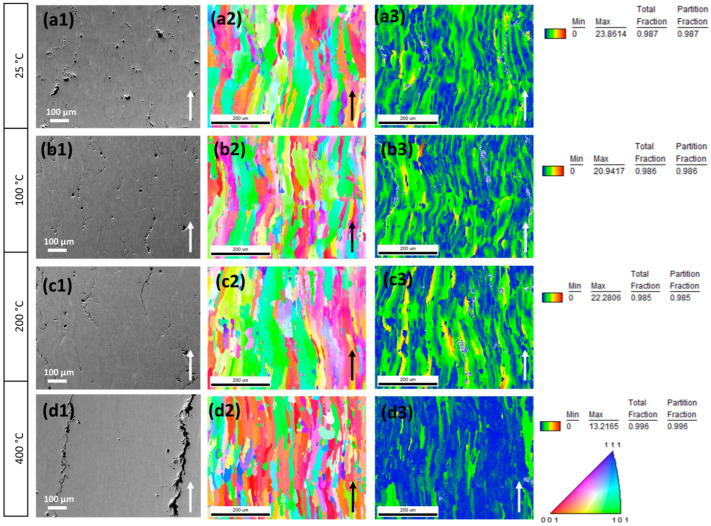
SEM images (**a1**–**d1**) of samples produced at 25, 100, 200, and 400 °C with 300 W of laser power and 800 mm/s, EBSD IPF Z maps (**a2**–**d2**), and GROD maps (**a3**–**d3**) showing internal stresses. The arrows indicate the build direction.

SEM equipped with EDS and EBSD was employed for detailed crack characterisation. Cracks, grains, and local strain features were analysed to assess their morphology and formation mechanisms (Figure 5). EBSD and processing of grain reference orientation deviation (GROD) analyses were used. EBSD data (Figure 5) enabled characterisation of grain size, morphology, and distribution. The results revealed that preheating temperatures of 100 °C and 200 °C had minimal-to-no effect on grain size, shape, internal stresses, or distribution.

Despite variations in preheating conditions, the microstructural characteristics remained largely unchanged, indicating that lower cooling rates did not affect the alloy’s grain structure under these thermal conditions. On the other hand, results revealed notable differences between samples printed at 400 °C and those at lower preheating temperatures. Specifically, the smaller grains exhibited a different orientation along the build direction in the samples subjected to the higher preheating temperature.

Furthermore, GROD analysis enabled quantification of grain-boundary misorientation, which is crucial for assessing the degree of crystallographic deviation within the material. GROD results indicate lower local strain in samples produced at 100 °C compared to 200 °C. This slight difference suggests that the higher preheating temperature may promote dislocation accumulation, possibly due to increased thermal stress during solidification. Conversely, samples produced at 400 °C exhibit a smaller dislocation density. This finding indicates that the material relaxes at preheating temperatures of 400 °C and above.

**Figure 6:**
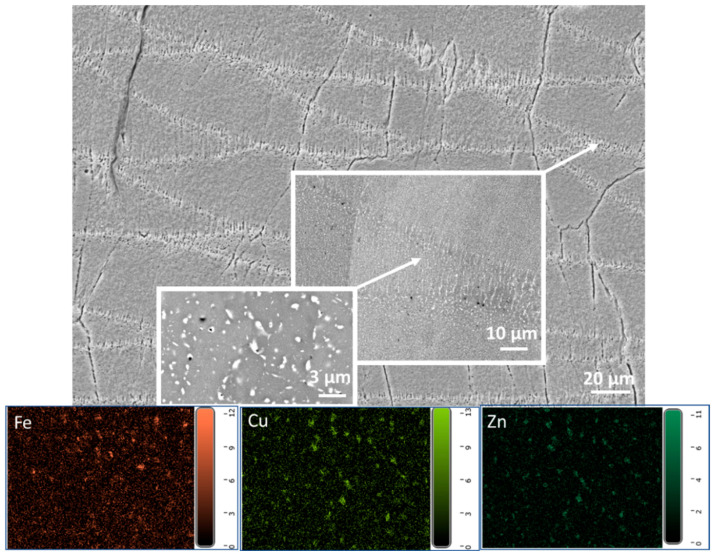
The main SE image shows intersecting cracks over melt pools, with clear precipitation visible along the melt pool boundaries. Below, EDS maps highlight the distribution of iron (Fe), copper (Cu), and zinc (Zn), coloured in orange, green, and blue, respectively.

The SE image in Figure 6 shows melt pools intersected by cracks. At the melt pool boundaries, precipitation is also visible. The EDS analysis results shown at the bottom of the image reveal the elemental distribution of Fe, Cu, and Zn at the melt pool boundary. This observation suggests that during the SLM process, these elements tend to concentrate at boundaries, potentially due to the rapid cooling rates typical of the process, leading to the formation of precipitates.

### 3.2. Hardness Measurements

Table 4 presents the results of Vickers hardness measurements (HV0.5) obtained on cross-sections perpendicular (⊥ BD) and parallel (‖ BD) to the build direction.

The sample fabricated without preheating (25 °C) exhibits an average hardness of 134 ± 2 HV0.5 in the ⊥ BD orientation and 96 ± 2 HV0.5 in the ‖ BD orientation. Increasing the preheating temperature to 100 °C yields the highest measured hardness, with values of 146 ± 3 HV0.5 (⊥ BD) and 103 ± 2 HV0.5 (‖ BD). Further increases in preheating temperature led to a reduction in hardness. At 200 °C, the hardness decreases to 139 ± 1 HV0.5 (⊥ BD) and 101 ± 3 HV0.5 (‖ BD), while a pronounced drop is observed at 400 °C, where the hardness reaches 117 ± 6 HV0.5 (⊥ BD) and 83 ± 7 HV0.5 (‖ BD).

The observed hardness trend correlates well with the internal stress state revealed by EBSD and GROD analyses, as described in Maleki et al. [19]. Since the loss of Zn and Mg was found to be independent of preheating temperature, as shown by Jadot et al. [18], the variation in hardness is primarily attributed to changes in dislocation density and thermal relaxation effects rather than to compositional differences. Moderate preheating at 100 °C appears to retain LPBF-induced strain hardening, whereas higher preheating temperatures promote recovery and stress relaxation, resulting in a reduced hardness response.

### 3.3. Thermal Simulations

**Figure 7:**
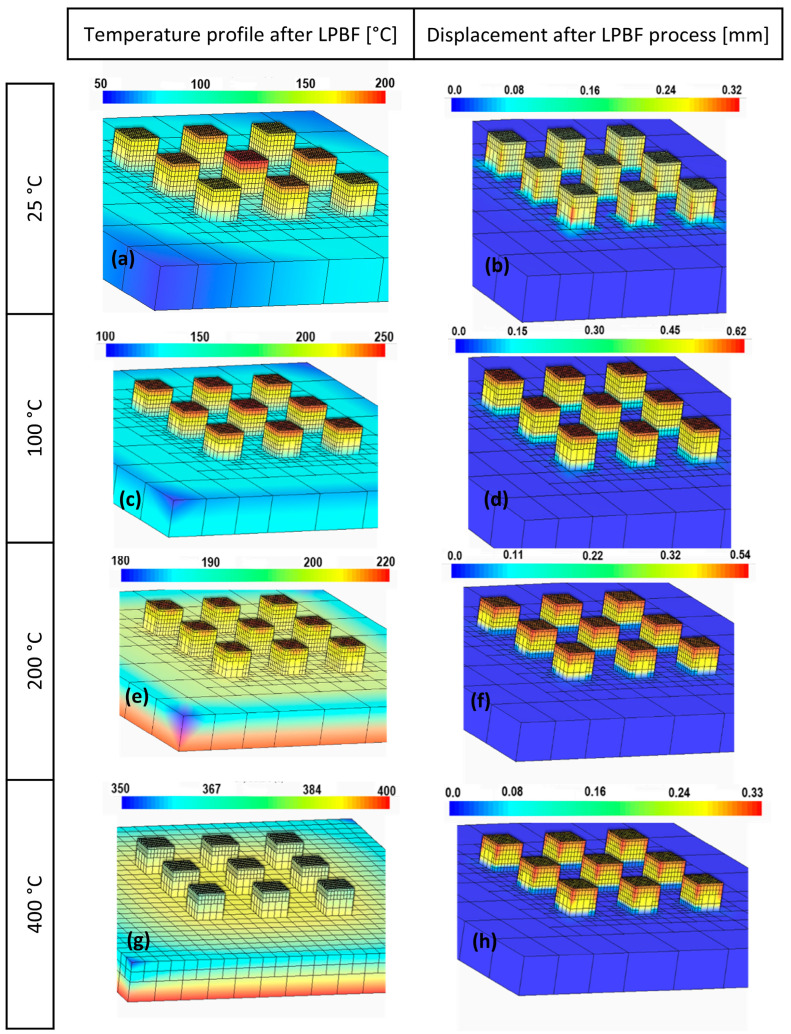
Simulation results: (**a**,**b**) represent states without preheating, (**c**,**d**) reflect the influence of preheating at 100 °C, (**e**,**f**) display the effects of preheating at 200 °C, and (**g**,**h**) depict the outcomes when preheating is set at 400 °C.

Figure 7 presents the thermal simulation results. The figure is divided into two columns showing thermal distribution and displacement. The left column of the figure illustrates the analytical heat distribution following the LPBF process. Colour mapping vividly portrays how heat is distributed within the sample. This data is a vital aspect of understanding the thermal behaviour during and after the LPBF process. At 25 °C preheating (Figure 7a), a gradual temperature gradient is observed across the part, with temperatures predominantly in the 50–100 °C range, indicating minimal residual heat after processing. At 100 °C, the temperature distribution is slightly higher, remaining within the 100–150 °C range, suggesting a moderate increase in retained heat that may affect the cooling rate and, consequently, the part’s properties. At 200 °C, the profile indicates a more pronounced temperature range within 180–220 °C, highlighting increased thermal retention that could significantly influence microstructure evolution. At 400 °C, the most intense thermal retention is observed, with temperatures in the 350–400 °C range. Displacement after the SLM process at 25 °C is below 0.16 mm, reflecting the standard cooling and solidification behaviour with lower preheating conditions. At 100 °C, there is a slight increase in displacement to about 0.22 mm, suggesting that preheating at this temperature begins to affect the material’s dimensional stability. At 200 °C, the displacement further increases to 0.34 mm, indicating that the elevated preheating temperature contributes to greater thermal expansion and possibly more significant thermal stresses. At 400 °C, the maximum displacement, peaking at 0.33 mm, aligns with the highest preheating temperature, indicating a significant influence of thermal stress and the material’s response to the elevated base temperature.

Figure 7 demonstrates the relationship between preheating temperature, thermal distribution, and displacement during the LPBF process. These results indicate the influence of preheating on thermal behaviour during additive manufacturing.

## 4. Discussion

The results of this study confirm that EN AW 7075 remains highly susceptible to hot cracking during LPBF processing, despite extensive parameter optimisation and moderate base plate preheating. Preheating up to 200 °C did not lead to significant changes in grain structure, crack morphology, or crack density, in agreement with earlier reports indicating that thermal input alone is insufficient to suppress hot cracking in high-strength 7xxx aluminium alloys [12,38]. The limited effectiveness of moderate preheating can be attributed to the fundamental metallurgical characteristics of EN AW 7075, including its wide solidification range, pronounced microsegregation, and high sensitivity to hot cracking [5]. Although preheating reduces thermal gradients to some extent, it does not sufficiently alter the rapid solidification conditions that govern crack initiation during LPBF. As a result, samples produced at 100 °C and 200 °C exhibit microstructural features and cracking behaviour comparable to those fabricated without preheating.

In contrast, preheating at 400 °C leads to the formation of distinct columnar crack networks, indicating a transition to a different cracking mechanism. At this elevated temperature, reduced thermal gradients and prolonged melt pool lifetimes likely promote enhanced segregation of low-melting Zn–Mg–Cu-rich phases and partial grain-boundary weakening [39,40]. These effects, combined with increased thermal expansion and displacement, facilitate crack propagation along the build direction. Importantly, EBSD-GROD analysis reveals that samples preheated to 400 °C exhibit lower internal stress levels compared to those processed at lower temperatures, suggesting that crack formation itself contributes to stress relaxation by releasing stored strain energy [41].

The hardness measurements further support this interpretation. While moderate preheating at 100 °C results in a slight increase in hardness, likely due to retained LPBF-induced strain hardening, a pronounced decrease in hardness is observed at 400 °C. Since the evaporation of Zn (~1 wt.%) and Mg (~0.3 wt.%) occurs independently of preheating temperature, the reduction in hardness at 400 °C is attributed primarily to thermal relaxation and reduced dislocation density rather than compositional changes. This demonstrates that stress relaxation at high preheating temperatures compromises mechanical integrity [42]. From an application perspective, the pronounced hardness reduction at 400 °C preheating further limits the industrial viability of this approach, as mechanical performance is compromised without eliminating cracking. The expanded hardness dataset further reveals a clear anisotropic response with respect to the build direction. Hardness measured perpendicular to the build direction is consistently higher than that measured parallel to the build direction across all preheating conditions, indicating the influence of LPBF-induced directional microstructure. The most pronounced reduction occurs at 400 °C, where hardness decreases to 117 ± 6 HV0.5 (⊥ BD) and 83 ± 7 HV0.5 (‖ BD), confirming substantial mechanical softening at elevated preheating temperature. The increased scatter at 400 °C further reflects the heterogeneous microstructural state associated with columnar crack networks and enhanced thermal relaxation.

Thermal simulations qualitatively corroborate the experimental observations, predicting increased thermal retention and displacement with increasing preheating temperature [15]. While preheating generally reduces residual stresses in many materials, the present results indicate that excessive preheating in EN AW 7075 induces alternative, temperature-assisted crack-growth mechanisms rather than improving printability. The increased thermal retention and displacement predicted at 400 °C correlate with the experimentally observed transition toward columnar crack networks, suggesting that prolonged melt pool lifetimes and enhanced thermal expansion contribute to temperature-assisted crack propagation.

Overall, the findings emphasise that the processing limitations of EN AW 7075 in LPBF are governed primarily by intrinsic metallurgical constraints, including solidification behaviour, hot cracking sensitivity, and the volatility of key alloying elements. Consequently, base plate preheating alone is insufficient to enable crack-free fabrication of this alloy. Future strategies should therefore focus on modified alloy chemistries, advanced laser-scanning strategies, or the development of crack-resistant derivative alloys rather than relying solely on thermal management.

## 5. Conclusions

This study investigated the applicability of laser powder bed fusion to the high-strength aluminium alloy EN AW 7075, with particular emphasis on the effect of base plate preheating.

The main conclusions can be summarised as follows:

- EN AW 7075 remains highly susceptible to hot cracking during LPBF processing, and preheating the base plate alone does not improve its printability
- Chemical analysis revealed consistent evaporation of Zn (~1 wt.%) and Mg (~0.3 wt.%) during LPBF, independent of the applied preheating temperature, indicating that compositional changes are primarily governed by melt pool conditions.
- Preheating at 100 °C and 200 °C did not significantly affect grain structure or crack morphology compared to samples produced without preheating.
- Preheating at 400 °C led to the formation of distinct columnar crack networks and pronounced stress relaxation; however, this stress reduction came at the expense of mechanical integrity, as evidenced by a significant decrease in hardness.
- Thermal simulations qualitatively supported the experimental observations, predicting increased thermal retention and displacement with increasing preheating temperature.

Overall, the results demonstrate that the processing limitations of EN AW 7075 in LPBF are governed by intrinsic metallurgical constraints, including solidification behaviour, hot-tearing sensitivity, and the volatility of key alloying elements. Consequently, alternative approaches such as alloy modification or advanced processing strategies are required to enable crack-free fabrication.

## Figures and Tables

**Table 1:** Chemical composition of the received EN AW 7075 powder in wt. [%].

	Zn	Cu	Cr	Fe	Mn	Ti	Si	Mg	Al
As received EN AW 7075	5.6	1.4	0.22	0.33	0.10	0.01	0.12	2.4	bal.

**Table 2:** Parameters used for sample manufacturing.

Sample Number	Laser Power (*P*) [W]	Scanning Speed (*v*) [mm/s]	Laser Beam Diameter (*d*) [µm]	Hatching Distance (*h*) [µm]	Layer Thickness (*t*) [µm]	Energy Density (*E*) [J/mm^3^]
**1**	300	600	60	60	30	277.7
**2**	400	1000	60	60	30	222.2
**3**	300	800	60	60	30	208.3
**4**	200	600	60	60	30	185.2
**5**	300	1000	60	60	30	166.7
**6**	200	800	60	60	30	138.9
**7**	300	1200	60	60	30	138.9
**8**	300	1400	60	60	30	119.1
**9**	150	800	60	60	30	104.2

**Table 3:** Chemical composition in wt.% (ICP analysis) of the as-received powder and after the LPBF process, with and without preheating involved.

	Zn	Cu	Cr	Fe	Mn	Ti	Si	Mg	Al
As-received EN AW 7075	5.6	1.5	0.22	0.33	0.10	0.02	0.12	2.4	bal.
After SLM at 25 °C	4.3	1.5	0.22	0.33	0.10	0.02	0.12	2.1	bal.
After SLM at 100 °C	4.5	1.5	0.21	0.33	0.10	0.02	0.12	2.1	bal.
After SLM at 200 °C	4.4	1.5	0.22	0.33	0.10	0.02	0.12	2.1	bal.
After SLM at 400 °C	4.5	1.5	0.22	0.33	0.10	0.02	0.11	2.1	bal.

**Table 4:** EN AW 7075 sample hardness after LPBF process.

Preheating T [°C]	Average Hardness [HV0.5] ⊥ BD	Average Hardness [HV0.5] ‖ BD
25	134 ± 2	96 ± 2
100	146 ± 3	103 ± 2
200	139 ± 1	101 ± 3
400	117 ± 6	83 ± 7

## Data Availability

The datasets supporting the findings of this study, including raw hardness measurements, chemical analysis data, microstructural images, and thermal simulation outputs, are available in the Zenodo repository at https://zenodo.org/records/15124437, accessed on 26 January 2026.
